# The case for investment in nutritional interventions to prevent and reduce childhood and adolescent overweight and obesity in Peru: a modelling study

**DOI:** 10.1186/s12966-024-01677-5

**Published:** 2024-11-06

**Authors:** Maria Elena Ugaz, Christina L. Meyer, Angela M. Jackson-Morris, Daphne Wu, M. Michelle Jimenez, Carlos Rojas-Davila, Carlos Orlando Zegarra Zamalloa, Elizabeth F. Ludwig-Borycz, D’Arcy Williams, Jo Jewell

**Affiliations:** 1 UNICEF Peru, Melitón Porras 350, Lima, 15074 Peru; 2 RTI International, 3040 East Cornwallis Road, P.O. Box 12194, Research Triangle Park, NC 27709-2194 USA; 3 University of Michigan School for Environment and Sustainability, 440 Church St., Ann Arbor, MI 48109 USA; 4 UNICEF, 125 Maiden Lane, New York, NY 10038 USA

**Keywords:** Obesity, Overweight, Economics, Peru, Adolescents, Children, Interventions, Investment

## Abstract

**Background:**

Between 2006 and 2016 the prevalence of overweight and obesity among children and adolescents aged 5–19 years in Peru increased from 22.7 to 27.0%. This investment case quantifies the economic impacts of childhood and adolescent overweight and obesity in Peru. It identifies and quantifies the potential impact of a set of new or expanded interventions that can strengthen current national efforts to prevent and reduce child and adolescent overweight and obesity.

**Methods:**

A deterministic Markov cohort model with a societal cost perspective estimated reductions in mortality and morbidity from implementing interventions to prevent and reduce child and adolescent overweight and obesity and the impact in savings in healthcare costs and gains in wages and productivity. Interventions identified through a review of published literature includes a school-based social marketing campaign, exclusive breastfeeding promotion and support, a healthy food and drink policy for school premises, and a 20% subsidy on fruits and vegetables for people living below the national poverty line. The return on investment (ROI) was calculated along with the estimated cost savings associated with the interventions. Analysis was conducted to test ROI sensitivity to changes in the key parameters and assumptions.

**Results:**

Between 2025 and 2092, the expected combined direct and indirect healthcare costs attributable to child and adolescent overweight and obesity in Peru are 210.6 billion USD. The direct healthcare costs are 1.8 billion USD, and the indirect costs are 208.8 billion USD. Expected savings for all interventions combined is 13.9 billion USD with a per-person savings of 12,089.8 USD. The expected ROI of the four interventions combined is 39.3 USD (30-years), 64.6 USD (50-years), and 164.1 USD (66-years) per one USD invested.

**Conclusions:**

The overweight and obesity epidemic among children and adolescents in Peru requires wide-ranging and expanded implementation of policies to achieve long-term reductions in prevalence. This study’s findings show that the four priority interventions have high ROIs and can be used to guide policy to address the complex interplay of factors that contribute to the obesogenic environment.

**Supplementary Information:**

The online version contains supplementary material available at 10.1186/s12966-024-01677-5.

## Background

Between 2006 and 2016 the prevalence of overweight and obesity among children and adolescents aged 5–19 years in Peru increased by from 22.7 to 27.0% [1]. Children and adolescents who are affected by overweight and obesity are at increased risk of developing high blood pressure, high cholesterol, type 2 diabetes, asthma, joint problems, gallstones, anxiety, and depression [2–5]. They tend to miss more school days and are at an increased risk of psychosocial difficulties while at school [6, 7]. Children and adolescents living with overweight and obesity are at increased risk of maintaining that status into adulthood [8]. Overweight and obesity in adulthood is also associated with increased risk for multiple non-communicable diseases (NCDs), such as cancer, cardiovascular disease (CVD), diabetes, Alzheimer’s disease, and asthma [9]. These overweight and obesity-attributable diseases account for 9% of premature deaths globally [10, 11]. In addition to the healthcare costs associated with treating these overweight and obesity-attributable diseases, economic impacts of overweight and obesity result from increased absenteeism (missed days of work), presenteeism (reduced productivity while at work), hiring discrimination, unemployment, lower income, disability, and premature death [12–14].

In middle-income countries (MICs), like Peru, that continue to deal with infectious diseases and undernutrition, the growing prevalence of overweight and obesity adds a double burden of malnutrition [15]. Moreover, the health and economic burdens are not equitably distributed within the population, with people in lower socioeconomic groups being at increased risk for overweight and obesity and related NCDs such as cancer and CVD [16]. The health and economic impacts of overweight and obesity are substantial at both the individual and societal level and represent an important health equity issue.

As children and adolescents aged 0–19 years are projected to make up 32% of Peru’s population in 2025—approximately 10.9 million children and adolescents [17], the Government of Peru has developed policies to address the factors that contribute to overweight and obesity among children and adolescents. In 2013, the government passed the Healthy Eating Promotion Law (Law No. 30021), which included a suite of interventions such as nutritional education and physical activity in schools, recommendations to provide healthy meals in school cafeterias and health centers, front-of-pack nutrition labeling, and restricted marketing of unhealthy foods [18]. However, rules for implementing the law were not approved until 2017 and the subsequent implementation of the law by the government has been slow [19, 20]. Further legislation in 2018 enacted tiered sugar-sweetened beverage (SSB) taxation, which was modified in 2021 to the current level of 25% for SSBs with more than five grams of sugar per 100 ml and 17% tax for SSBs with sugar content between 0.5 and five grams per 100 ml [21]. This tiered design incentivized the beverage industry to reduce the sugar content of SSBs [22]. Even so, overweight and obesity prevalence among children has continued to rise in Peru, increasing in children under 5 years from 6.4% in 2019 to 7.8% in 2021, demonstrating that further measures are necessary to address the obesogenic environment and reduce the prevalence of overweight and obesity and their sequelae [23].

This paper reports the results of an investment case for childhood and adolescent overweight and obesity in Peru. We aim to quantify the health and economic impacts of childhood and adolescent overweight and obesity in Peru and the potential gains from a set of new or expanded interventions. The investment case identifies which interventions will generate the largest health and economic returns with the goal of supporting resource allocation and prioritization to efficiently respond to the increasing challenge of childhood and adolescent overweight and obesity.

## Methods

This investment case applies a methodology that was developed and piloted in Mexico to assess the health and economic impact of child and adolescent overweight and obesity prevention and reduction interventions [24]. The model is a deterministic Markov cohort combining the Assessing Cost-Effectiveness in Obesity (ACE-Obesity) and the Early Prevention of Obesity in Childhood (EPOCH) models [25, 26]. We estimated reductions in mortality and morbidity from interventions and the resulting economic impact in terms of averted mortality, savings in healthcare costs, and gains in wages and productivity. The model applies a societal cost perspective that includes all costs and health effects regardless of the payer or beneficiary, providing insight into the impact interventions will have on the wider economy and society [27, 28]. The model cohort includes children and adolescents aged 0–19 years in 2025 and estimates impacts until 2092, when the mean age of the cohort will be 77 years – the current Peruvian life expectancy [17]. 2025 was selected as the base year to allow time to prepare and implement interventions. Results are presented in 2020 USD dollars (USD) and Peruvian sol (SOL) using exchange rate and consumer price index data from the World Bank. The analysis was conducted in Microsoft Excel according to the Consolidated Health Economic Evaluation Reporting Standards’ practice guidelines (Table S1) [29]. In the absence of similar studies to validate the model against, the model’s code was reviewed by an analyst who was not involved in creating the model. A summary is provided in the sub-sections below with additional details in the **Supplementary Materials**.

### Baseline scenario

The baseline scenario estimated the health and economic outcomes attributable to overweight and obesity in children and adolescents aged 0–19 years in 2025 (the model cohort) throughout their lifetime. We assumed that current trends in mortality, morbidity, and risk factors in Peru remain unchanged and projected future mean BMI and overweight and obesity prevalence, disaggregated by age and sex.

The projected prevalence of overweight and obesity was used to calculate future health and economic impacts. First, we estimated the years of life lost (YLLs), years lived with disability (YLDs), and disability-adjusted life-years (DALYs) attributable to overweight and obesity in the model cohort. We discounted the effects at 3% annually, the standard discount rate for global health economics research [28, 30, 31], and used the standard discounting formula to calculate the all-cause YLLs for age group by year to reflect the social preference of a healthy year now rather than in the future [32]. Future overweight and obesity-attributable YLLs were estimated using the same proportion of all-cause YLLs due to overweight and obesity-attributable diseases or conditions [33, 34] obtained from projected all-cause and cause-specific mortality rates from 2026 to 2092 using an autoregressive integrated moving average (ARIMA) model [35]. The analysis uses Peru’s gross domestic product (GDP) per capita (6,064 USD in 2020) as a proxy for the value of a life year to capture economic losses from premature mortality [31]. We then calculated the YLDs for each overweight and obesity-attributable condition within the 5-year age groups [11, 36]. Finally, the economic impact of children and adolescents affected by overweight and obesity was estimated at baseline by the combination of healthcare costs attributable to obesity (excluding overweight) during childhood and adulthood, and the impact that overweight and obesity has on future labor productivity.

### Obesity-attributable healthcare cost

Data on additional healthcare costs is only available for children and adolescents affected by obesity; therefore, the analysis includes only the additional healthcare costs of obesity (excluding overweight). To obtain obesity-attributable healthcare costs, we multiplied the number of individuals affected by obesity, by age group, by the incremental annual healthcare expenditure for a person affected by obesity. Annual healthcare expenditure was calculated by multiplying the average annual healthcare expenditure per person by the incremental percentage of higher healthcare expenditure paid by individuals affected by obesity (as compared to those of healthy weight), disaggregated by age [37, 38]. Average annual healthcare expenditure in Peru was obtained from the WHO Global Health Expenditure Database [39]. All costs were inflated to 2020 Peruvian Sol values using the consumer price index (CPI) [30, 31, 40].

### Impact on labor productivity

Productivity losses due to childhood and adolescent overweight and obesity were modeled for lifetime wage loss due to lower education attainment and productivity loss due to absenteeism and presenteeism [41]. To estimate the impact on educational attainment, the number of individuals affected by overweight and obesity at age 17 (the age when individuals in Peru complete secondary education to begin tertiary education) and the proportion of the Peruvian population aged 25–34 with tertiary education, were used to estimate the number of individuals who would not attain tertiary education due to overweight and obesity [42]. We accounted for the wages earned during the five additional years that individuals who do not attend tertiary school may have worked (since tertiary education lasts for five years in Peru) and assumed that every individual who attained tertiary education and participates in the workforce would enter the workforce at age 22 and exit at age 65, the legal retirement age under the Peruvian pension system [43].

### Intervention scenario

To identify and select priority interventions we focused not only on the costs and cost-effectiveness of interventions aimed at preventing and reducing childhood and adolescent overweight and obesity, but also on the cultural and policy environment in Peru. We conducted Peru-specific and global literature reviews of the costs and cost-effectiveness of interventions for childhood and adolescent overweight and obesity. The search also identified baseline coverage levels of selected interventions from published and grey literature sources. Additionally, we used purposive sampling to identify key informants (*n* = 4) from the Peruvian government, academia, research institutes, and civil society working in the areas of nutrition, public policy, and addressing overweight and obesity. Utilizing a semi-structured interview guide (see Supplementary Exhibit S1), semi-structured interviews were conducted in Spanish and English. Content analysis was employed to distil interviewees’ views regarding the interventions they considered most valuable and applicable to the Peru context [44, 45].

The following four priority interventions were identified [46, 47]. First, implementing a social marketing campaign focused on behavioral nudges in schools focused on healthy diets and physical activity with the involvement of parents and caregivers. Second, as Peru’s exclusive breastfeeding rate for infants under six months was 68.4% in 2020, promoting and providing support for exclusive breastfeeding in the first six months via individual counseling, group education, breastfeeding support at delivery, and lactation management in antenatal and prenatal care, hospitals, and delivery centers [48]. This would also entail wider community-based group counseling and education. Third, scaling up the policy only allowing healthy food and drink on school premises policy currently in place in Lima to the rest of the country, as well as raising awareness among staff, managers, parents, and students [49]. Last is the introduction of a 20% fruit and vegetable subsidy for people living below the national poverty line. The subsidy on fruit and vegetables, social marketing in schools, and breastfeeding promotion and support have not been previously implemented in Peru, while the healthy school food environment interventions have previously only been implemented in Lima. Evidence on the effect sizes in terms of BMI or overweight and obesity prevalence reduction, as well as the unit costs associated with each of the interventions were obtained from the literature **(**Table 1**)**. Due to limited Peru-specific data and studies, the analysis applies evidence from wider Latin American countries and the global literature.

**Table 1:** Baseline level of interventions in Peru and Effect size and cost data to Reach Target goals

Intervention	Target population	Baseline coverage	Target coverage	Effect size (95% CI)	Effect size source	Unit cost (cost per child, 2020 USD)	Cost components	Unit cost source
Social marketing in schools	Children and adolescents aged 6–16 years	0%	80% of target population	-0.25 (-0.45, -0.04) BMI reduction	Aceves-Martins et al. (2016) [66]	USD 1.04	Program organization costs, training of teachers and food service staff, extra teaching, and additional curricular activities, such as brochures and books	Cecchini et al. (2010) [67]
Breastfeeding promotion at health centers	Mothers of infants aged 6 months or less	0%	85% of the target population	Average 5.2% (3.5%, 6.5%) reduction in obesity prevalence at age 5	Rollins (2016)[53] and Holla-Bhar (2015)[68]	USD 21.57	Health education to mothers and training health workers and community volunteers	Rollins (2016)[53] and Bhutta et al. (2013)[69]
Healthy school food environment	Children and adolescents aged 6–16 years	3.38%	80% of target population	Boys: 5.1% (0.9%, 9.3%) reduction in overweight prevalence Girls: 1.8% (-2.8%, 6.4%) reduction in overweight prevalence (Not significant)	Levasseur (2021)[70]	USD 0.19	Basic administration, planning, enforcement, preparation and distribution of posters, and monitoring	Sassi (2010)[71]
Targeted 20% food subsidy for population under the national poverty line	Children and adolescents aged 0–19 years	0%	20.2% of national population (Figure based on proportion of children and adolescents living below the poverty line).	-0.08 (-0.16, 0.00) BMI reduction	Afshin et al. (2017)[65]	USD 0.02	Planning and development, operations, administration, and monitoring	Sassi (2010)[71]

The costs of implementing the interventions were applied from 2025 to 2044, the year when the last subcohort would turn 19 years old. We used a baseline coverage of 0% for interventions not previously implemented in Peru (social marketing interventions in schools, breastfeeding promotion and support, and the subsidy on fruits and vegetables). While the prevalence of exclusive breastfeeding in Peru is currently 68.4% and individual breastfeeding promotion efforts exist throughout the country, we modeled a coordinated national breastfeeding promotion policy to improve the design, implementation, and coverage of Baby Friendly Hospital Initiative (BFHI) and breastfeeding promotion through community health workers. For the national baseline coverage of the healthy school food environment, we first calculated the number of students in primary school (aged 6–11) and secondary school (aged 12–16) in Lima by multiplying the population of primary school and secondary school age in Peru by the school enrollment ratio for primary and secondary schools, respectively, and the proportion of the population of Peru living in Lima. Population, by sex, in Peru was obtained from the United Nations’ World Population Prospects (WPP) [17], school enrollment ratio was obtained from the World Bank’s World Development Indicators database [50], and the proportion of the Peruvian population living in Lima was based on the 2017 Peru census [51].

The target coverage level was set at 80% for the school-based social marketing and the healthy school food environment and 20.2% for the fruit and vegetable subsidy, which is the percentage of the population in Peru that lives under the national poverty line. For breastfeeding promotion and support, we aimed to increase the prevalence of exclusive breastfeeding from birth to six months from the current 68.4–85% [52]. To estimate the coverage level for BFHI and community intervention required to reach the target exclusive breastfeeding prevalence of 85%, we used the relative risks (RR) of exclusive breastfeeding from birth to six months with health systems and community interventions from Rollins et al., 2016 [53]. Based on this RR, we set the target coverage level for BFHI and community intervention at 16%. For the target population, we selected children and adolescents aged 0–19 years for the fruit and vegetable subsidy (the subsidy would go to the head[s] of household), children and adolescents 6–16 years (primary and secondary school age in Peru) for the school-based social marketing and healthy school food environment interventions, and children aged under one year in 2025 for the breastfeeding promotion intervention.

We assumed that intervention effects are realized one year after implementation [54]. We then estimated the impact of the interventions on YLLs, YLDs, and DALYs saved, healthcare costs averted, productivity gained, and lifetime wages gained due to increased educational attainment. We calculated the effect of each intervention on baseline BMI of the model cohort for each age and sex group. We assumed that the change in BMI was maintained into adulthood and estimated the resulting reduction in projected overweight and obesity prevalence by sex and age using the potential impact fraction (PIF) to calculate the reductions in mortality and morbidity [24].

### Indicator

The investment case examines the efficiency of the interventions through a return-on-investment (ROI) analysis. The ROI (or benefit-cost ratio) is calculated by dividing the total economic value gained from the interventions by the cost to implement the interventions. The ROI analysis compares the cost of implementation to all economic benefits – averted mortality, healthcare costs averted, and wages and productivity gained. The analysis reviews this indicator of efficiency over 30 years, 50 years, and lifetime horizons in order to capture medium and long term costs and benefits of the intervention [53].

### Sensitivity analysis

Additional analyses were conducted to test the sensitivity of the results to changes in the assumptions. First, a 95% confidence interval (95% CI) for the ROI estimates was derived by utilizing the upper and lower limits of the intervention effect size reported in the literature (Table 2). Second, the effects of interventions were modelled to be realized two years after implementation instead of one year after implementation (Supplementary Table S3). Third, the impact of childhood and adolescent obesity on education attainment were varied such that obesity in childhood has been associated with a 43% lower likelihood of not completing 12 or more years of education [42, 55]. Fourth, sensitivity analyses were conducted to consider how changes in national income (GDP per capita) impact upon the value of these changes in mortality or life expectancy. As such, the valuation of a life year was increased by independently multiplying GDP per capita by a global GDP multiplier of 1.6 and a regional GDP multiplier for Latin America of 1.4 [12, 56]. An additional sensitivity analysis substituted annual discount rate of 3% with 5% to illustrate a preference for receiving benefits earlier [57]. Finally, as the main analysis estimates the combined ROI of implementing the four interventions by assuming the impact of each intervention is additive and independent, a sensitivity analysis was conducted to examine the ROI results of a less-than-additive impact and a more-than-additive impact by estimating the ROI of the combined intervention package if each intervention’s impact was reduced by 50% and increased by 10% [24].

**Table 2:** Economic gains resulting from the four interventions, 2026–2092

	Healthcare cost savings	Gains from increased wages	Productivity gained from averted work absenteeism and presenteeism	Economic value of life years gained (YLLs only)	Total savings	Total savings per person affected by overweight or obesity
Intervention	UDS millions					USD
Social marketing in schools	93.4 (14.9, 168.2)	112.7 (18.0, 202.8)	250.0 (40.0, 450.0)	955.5 (152.9, 1,719.8)	1,411.5 (225.8, 2,540.8)	9,329.9 (9,035.9, 9,609.9)
Breastfeeding promotion at health centers	33.9 (22.9, 42.6)	49.8 (33.7, 62.6)	83.9 (56.8, 105.5)	278.0 (188.3, 349.7)	445.6 (301.8, 560.5)	4,355.8 (4,323.0, 4,382.1)
Healthy school food environment	845.3 (683.6, 1,007.0)	784.0 (645.9, 922.2)	2,449.9 (1,989.9, 2,910.0)	7,823.8 (6,506.2, 9,141.4)	11,903.1 (9,825.7, 13,980.5)	11,775.7 (10,860.4, 12,583.2)
20% food subsidy	5.3 (0.0, 16.2)	6.0 (0.0, 18.5)	14.0 (0.0, 43.2)	54.0 (0.0, 166.2)	79.3 (0.0, 244.1)	5,273.7 (0.0, 5,314.7)
All four combined	977.8 (721.5, 1,233.9)	952.5 (697.7, 1,206.1)	2,797.9 (2,086.7, 3,508.7)	9,111.3 (6,847.4, 11,377.1)	13,839.5 (10,353.3, 17,325.9)	12,089.8 (10,895.3, 13,135.4)

## Results

Table 3 shows the cost of implementation for each intervention independently and combined.

**Table 3:** Implementation costs

Implementation cost	SOL (millions)	USD (millions)
Social marketing in schools	256.93	73.51
Breastfeeding promotion at health centers	3.90	1.12
Healthy school food environment	32.25	9.23
20% food subsidy	0.76	0.22
All four combined	293.84	84.08

In Table 4 the base-case scenario health outcomes for the model cohort are given in lost YLLs, YLDs, and DALYs attributable to childhood and adolescent overweight and obesity. Overall, childhood and adolescent overweight and obesity in Peru causes 15.2 million YLLs, 6.5 million YLDs, and 21.7 million DALYs from 2026 to 2092. These results differ by sex, with more YLLs, YLDs, and DALYs resulting from childhood and adolescent overweight and obesity among males than females.

**Table 4:** Health outcomes for the model cohort in the base-case scenario, 2026–2092

	Years of life lost (YLLs)	Years of healthy life lost due to disability (YLDs)	Disability adjusted-life years (DALYs)
Males	8,169,214	3,292,040	11,461,254
Females	7,020,499	3,179,563	10,200,061
Total	15,189,712	6,471,603	21,661,315

The total combined direct healthcare costs and indirect costs attributable to child and adolescent overweight and obesity in Peru are 210.6 billion USD (736.0 billion SOL) **(**Table 5**)**. Direct healthcare costs account for just 1% of the total cost (1.8 billion USD), while indirect costs are the large majority of costs (208.8 billion USD). Indirect costs include loss in lifetime wages of 0.9 billion USD, productivity loss of 16.9 billion USD, and mortality costs of 190.9 billion USD.

**Table 5:** Direct and indirect costs attributable to childhood and adolescent overweight and obesity, 2026–2090 total

	Total cost USD (billions)	Total cost SOL (billions)	Average lifetime cost per child affected by overweight or obesity (USD)
Direct healthcare costs (obesity only)			
During childhood	0.2	0.6	190.8
During adulthood	1.7	5.8	1,981.3
Total direct healthcare costs	1.8	6.3	2,172.1
Indirect costs			
Loss in lifetime wages	0.9	3.2	332.5
Productivity loss	16.9	59.2	20,277.0
Mortality costs	190.9	667.3	69,942.9
Total indirect costs	208.8	729.7	76,481.4
Total costs (direct and indirect)	210.6	736.0	77,146.2

The lifetime reduction of DALYs attributable to the interventions are 124.6 thousand for the social marketing intervention in schools, 38.0 thousand for breastfeeding promotion and support at health centers, 942.9 thousand for the healthy school food environment intervention, 7.0 thousand for the food subsidy, and 1.1 million for all four interventions combined (Table 6**).**

**Table 6:** Impact of interventions on DALY reductions during the lifetime of the model cohort

Intervention	Lifetime reduction DALYs (95% CI, thousands)
Social marketing in schools	124.6 (19.9, 224.3)
Breastfeeding promotion at health centers	38.0 (25.7, 47.9)
Healthy school food environment	942.9 (783.6, 1,102.3)
20% food subsidy	7.0 (0.0, 21.7)
All four combined	1,112.7 (829.3, 1,396.2)

Total lifetime savings for all four interventions combined is 13.8 billion USD with a per-person savings of 12,089.8 USD **(**Table 2**)**. The economic benefits were expected to equal the implementation costs 18 years after implementing the combined interventions. The economic gains resulting from the four interventions come from healthcare cost savings of (977.8 million USD, gains from increased wages (952.5 million USD), productivity gained from averted work absenteeism and presenteeism (2.8 billion USD), and economic value of life years gained (9.1 billion USD). The disaggregated total savings by intervention are 1.4 billion USD from the social marketing intervention in schools, 445.6 million USD from breastfeeding promotion and support at health centers, 11.9 billion USD from the healthy school food environment intervention, and 79.3 million USD from the food subsidy.

Table 7 shows the return-on-investment (ROI) of the four interventions independently and combined. The ROI of the four interventions combined is 39.2 USD (30 years), 64.4 USD (50 years), and 163.6 USD (lifetime) per one USD invested. The healthy school food environment intervention would have the greatest ROI across all three-time horizons with an ROI of 325.1 USD (30 years), 518.1 USD (50 years), and 1,289.1 USD (66 years; lifetime).

**Table 7:** Return-on-investment (ROI) of selected childhood and adolescent obesity interventions over a 30-year, 50-year, and lifetime time horizon

	ROI (USD)		
Intervention	Over 30 years	Over 50 years	Over lifetime
Social marketing in schools	2.9 (-0.4, 6.1)	6.3 (0.2, 12.1)	18.2 (2.1, 33.6)
Breastfeeding promotion at health centers	53.8 (36.1, 67.9)	130.0 (87.7, 163.8)	397.8 (269.2, 500.8)
Healthy school food environment	325.1 (275.3, 374.8)	518.1 (437.2, 599.1)	1,289.1 (1,063.9, 1,514.2)
20% food subsidy	81.5 (-12.1, 176.6)	154.2 (-21.9, 333.3)	380.6 (-52.4, 820.9)
All four combined	39.3 (30.4, 48.0)	64.6 (49.3, 79.6)	164.1 (122.1, 205.1)

Table S3 shows the ROIs from the sensitivity analyses. The interventions’ effects being realized two years after implementation, as opposed to one year, resulted in moderately lower ROIs for all interventions except the healthy school food environment intervention which was significantly decreased. In this conservative scenario, the 20% subsidy had the highest ROI. Using an annual discounting rate of 5% resulted in lower ROIs across all time horizons and interventions. Increasing the impact of childhood and adolescent obesity on education attainment, valuing one life-year as GDP per capita times the global GDP multiplier (1.6), valuing one life-year as GDP per capita times the regional GDP multiplier for Latin America (1.4), and assuming the combined package of interventions had a more-than-additive impact all slightly increased the ROIs across all time horizons and interventions. If the intervention package had a less-than-additive impact, the package of interventions would still offer Peru a positive ROI within a 30-year time horizon.

## Discussion

This analysis identified the significant health and economic impacts (direct and indirect) of child and adolescent overweight and obesity in Peru. The estimated lifetime costs from child and adolescent overweight and obesity included in this analysis is 24.3 times greater than Peru’s annual government health spending in 2020 [58]. The four interventions examined in the investment case—a social marketing campaign in schools, promotion and support for breastfeeding at health centers, a policy advocating for a healthy food environment in schools, a 20% food subsidy—all yield positive returns on investment. Of the interventions, the policy mandating availability of only healthy food and drinks for students at school demonstrates the highest ROI. Together the interventions provide economic gains of 13.8 billion USD over a 66-year period, or an annual average gain of 209.7 million USD, equivalent to 2.4% of Peru’s annual government health spending in 2020 [58]. Savings incurred by implementing all four interventions over a lifetime are equal to 1.6 times Peru’s government health expenditure in 2020 [58].

The high economic gains from implementing the interventions are likely due to the interventions altering the obesogenic environment, rather than relying solely on education or awareness-raising alone [59]. The intervention that independently has the highest lifetime health gains and ROI is the national healthy school food environment intervention to implement a policy requiring that only healthy food and drinks are available to students at school. Traditional school-based interventions have been shown to be limited in their capacity to improve health and economic outcomes when they focus solely on education [25]. The findings from this analysis shed light on how the impact that school-based interventions can have may be magnified by addressing part of the obesogenic environment within schools by ensuring only healthy food and drinks are available. The healthy school food environment intervention would expand Lima’s municipal law requiring schools to provide only healthy food and drinks in and around schools to the whole country, thereby broadening the scope of the national Health Eating Promotion Law [49]. An evaluation of the current compliance with the policy in Lima schools found that participating schools had less than 40% compliance [60]. Hence the proposed national scale-up of the local policy would also include awareness-raising with school staff, managers, parents, and students, as well as enforcement. Specific consideration to enforcement and education will likely require the most attention in order to ensure successful implementation.

The breastfeeding promotion and support intervention offers the second highest ROI with a lifetime ROI of 397.8. This intervention will complement the existing national ‘Comprehensive Maternal Health Care’ policy that recommends prenatal check-ups at primary care centers include vaccinations, vital measurements, blood tests, fetal imaging, and education about the six signs of pregnancy complications and six health and lifestyle topics (including breastfeeding) [61, 62]. It also complements Peru’s implementation of the International Code of Marketing of Breast-Milk Substitutes. A recent evaluation of Peruvian prenatal care found that only about half of women who attend their prenatal visits currently receive information about breastfeeding [63]. Therefore, this intervention will provide additional support during prenatal visits to ensure women receive information about breastfeeding. It will also promote exclusive breastfeeding at hospitals, delivery centers, and in the community through a mix of counseling, group education, and mass media campaigns. As these components would be new to the Peruvian context at a national, coordinated level their implementation would likely benefit from a stakeholder readiness and capacity development assessment prior to scaling up the intervention’s coverage.

The third highest ROI is for the 20% fruit and vegetable subsidy for people on low incomes. The positive ROI is consistent with previous findings from an investment case in Mexico that identified fiscal interventions (including both subsidies and taxes) as offering a high ROI for reducing childhood and adolescent overweight and obesity [24]. In the present analysis, the fruit and vegetable subsidy was included as a standalone policy, as the Peruvian government has already implemented a substantial SSB tax [21]. The proposed Peru subsidy additionally focused on a narrower subset of the population – people living under the national poverty line, based on evidence suggesting that this more focused subsidy would be best in the Peruvian context [64]. It should also be noted that there is uncertainty regarding the ROIs and the health and economic impact of the fruit and vegetable subsidy due to the zero- or negative lower limits of the 95% CI. These results reflect that there is some degree of uncertainty in the effect of food subsidies on overweight and obesity owing to the currently underdeveloped state of evidence across a wider range of countries; nonetheless we opted to include the targeted fruit and vegetable subsidy as a potential option given the positive indications from Afshin, et al.’s 2017 systematic review and meta-analysis of the impact of food pricing on improving dietary consumption and indication of interest from stakeholders [65]. As this subsidy does not currently exist in Peru, further research will be needed to develop an implementation plan capable of effectively targeting only populations living below the national poverty line.

The sensitivity analysis scenario in which the interventions’ effect size are not realized until two years after implementation (instead of one) found that the highest ROI was for the 20% fruit and vegetable food subsidy to those living below the national poverty line. This conservative scenario did not substantially change the ROI for the 20% fruit and vegetable food subsidy because the impact is across all age groups in both the main analysis and the sensitivity analysis, whereas the impact from the healthy school food environment intervention changes from being in effect from age 7 in the main analysis to being in effect from age 8 in the sensitivity analysis. Nonetheless, each intervention, individually or combined as part of a package, routinely demonstrates strong impacts across all seven sensitivity analyses and would bolster the existing Peruvian policies to mitigate the obesogenic environment by making healthy food more affordable (through the subsidy) and more available (through school-based healthy food requirements).

A primary strength of the present study is that it is the first investment case on childhood and adolescent overweight and obesity in South America. Additionally, the study entailed close collaboration with national nutrition policy experts and government officials, as well as UNICEF Peru to create a set of intervention recommendations that are uniquely suited to the Peruvian context. The model applied conservative assumptions and estimates based on peer-reviewed intervention literature and examined the result sensitivity to changes in the key parameters and assumptions.

There are several limitations that should be considered. First, the estimated costs attributable to childhood and adolescent overweight and obesity are only limited to the cohort modeled and would be lower than population-level costs. Second, the model assumes that the interventions permanently changed children and adolescents’ BMI and longitudinal data would be needed to confirm this. Finally, the model assumes that intervention effect sizes are independent (the probability of one intervention does not impact the probability of another intervention occurring) and additive (the effect size of all four interventions combine is the sum of the four individual intervention effect sizes) as no data is currently available to indicate the potential interplay of multiple interventions being implemented simultaneously. However, as some interventions share implementation settings, such as the healthy school food environment and school-based social marketing campaign interventions, linking these interventions could potentially improve implementation cost efficiency.

In the face of persistent increases in overweight and obesity in Peru over recent years, it is likely that an expanded set of robust policy interventions will be necessary to achieve long-term progress in addressing the overweight and obesity epidemic among children and adolescents. This study’s findings can be used to guide policy and strategy to address the complex interplay of factors that contribute to the obesogenic environment.

## Conclusion

Without further intervention, child and adolescent overweight and obesity are expected to pose a significant burden on Peru’s long-term health and economy. Addressing this epidemic will require wide-ranging and expanded implementation of policies to achieve long-term reductions in prevalence. This study’s findings show that national interventions to establish healthy school food environments, support and promote breastfeeding, subsidize fruit and vegetable sales for low-income households, and implement social marketing campaigns with behavioral nudges in schools can offer Peru strong health and economic returns on investment, both individually and collectively. The high economic gains these interventions offer is likely due their ability to alter the obesogenic environment, rather than solely relying on education or awareness-raising alone. These findings offer an evidence-base to guide future policies and strategies to address child and adolescent overweight and obesity.

## Electronic supplementary material

Below is the link to the electronic supplementary material.

Supplementary Material 1

## Data Availability

The datasets used and/or analyzed during the current study are available in publicly accessible databases including the World Bank’s World Development Indicators (https://datatopics.worldbank.org/world-development-indicators/), the International Labour Organization’s ILOSTAT database (https://ilostat.ilo.org/), NCD Risk Factor Collaboration (https://ncdrisc.org/data-downloads.html), Global Burden of Disease Study (https://www.healthdata.org/gbd), and the UN World Population Prospects (https://population.un.org/wpp/).

## Abbreviations

ARIMA: Autoregressive integrated moving average
BFHI: Baby Friendly Hospital Initiative
CI: Confidence interval
CPI: Consumer price index
CVD: Cardiovascular disease
DALY: Disability–adjusted life year
GDP: Gross domestic product
MIC: Middle income country
NCD: Noncommunicable disease
OR: Odds ratio
PIF: Potential impact fraction
ROI: Return–on–investment
RR: Relative risk
SSB: Sugar sweetened beverage
SOL: Peruvian sol
USD: US dollar
WPP: World Population Prospects
YLD: Year of healthy life lost due to disability
YLL: Year of life lost

## References

[1] Prevalence of overweight among children and adolescents. BMI > + 1 standard deviations above the median (crude estimate) (%). [cited 2023 May 19]. https://www.who.int/data/gho/data/indicators/indicator-details/GHO/prevalence-of-overweight-among-children-and-adolescents-bmi-1-standard-deviations-above-the-median-(crude-estimate)-(-).

[2] Cote AT, Harris KC, Panagiotopoulos C, Sandor GGS, Devlin AM. Childhood obesity and cardiovascular dysfunction. J Am Coll Cardiol. 2013;62(15):1309–19. 23954339 10.1016/j.jacc.2013.07.042

[3] Bacha F, Gidding SS. Cardiac abnormalities in youth with obesity and type 2 diabetes. Curr Diab Rep. 2016;16(7):1–9. 27168062 10.1007/s11892-016-0750-6

[4] Morrison KM, Shin S, Tarnopolsky M, Taylor VH. Association of depression & health related quality of life with body composition in children and youth with obesity. J Affect Disord. 2015;172:18–23. 25451390 10.1016/j.jad.2014.09.014

[5] Beck AR. Psychosocial aspects of obesity. NASN School Nurse. 2016;31(1):23–7. 26739931 10.1177/1942602X15619756

[6] Li Y, Raychowdhury S, Tedders SH, Lyn R, Lòpez-De Fede A, Zhang J. Association between increased BMI and severe school absenteeism among US children and adolescents: findings from a national survey, 2005–2008. Int J Obes. 2012;36(4):517–23. 10.1038/ijo.2012.15 22349572

[7] Carey FR, Singh GK, Brown HS, Wilkinson AV. Educational outcomes associated with childhood obesity in the United States: cross-sectional results from the 2011–2012 National Survey of children’s Health. Int J Behav Nutr Phys Activity. 2015;12(1):1–11. 10.1186/1479-5868-12-S1-S3 PMC4663995 26222699

[8] Gordon-Larsen P, The NS, Adair LS. Longitudinal trends in obesity in the United States from adolescence to the third decade of life. Obesity. 2010;18(9):1801–4. 20035278 10.1038/oby.2009.451 PMC2929301

[9] World Health Organization. Obesity and Overweight fact sheet. Geneva, Switzerland: World Health Organization. 2021. https://www.who.int/news-room/fact-sheets/detail/obesity-and-overweight

[10] World Health Organization. Obesity and overweight. [cited 2022 Nov 15]. https://www.who.int/news-room/fact-sheets/detail/obesity-and-overweight

[11] Global Burden of Disease Collaborative Network. Global Burden of Disease Study 2019. (GBD 2019) Results. Institute for Health Metrics and Evaluation. 2021 [cited 2022 Nov 20]. http://ghdx.healthdata.org/gbd-results-tool

[12] Okunogbe A, Nugent R, Spencer G, Ralston J, Wilding J. Economic impacts of overweight and obesity: current and future estimates for eight countries. BMJ Global Health. 2021;6(10). 10.1136/bmjgh-2021-006351 PMC8487190 34737167

[13] Campos-Vazquez RM, Gonzalez E. Obesity and hiring discrimination. Econ Hum Biol. 2020;37:100850. 31954211 10.1016/j.ehb.2020.100850

[14] Afshin AFM, Reitsma M. Health effects of overweight and obesity in 195 countries over 25 years. N Engl J Med. 2017;377(1):13–27. 28604169 10.1056/NEJMoa1614362 PMC5477817

[15] WHO. Double burden of malnutrition. [cited 2022 Nov 21]. https://apps.who.int/nutrition/double-burden-malnutrition/en/index.html

[16] Williams J, Allen L, Wickramasinghe K, Mikkelsen B, Roberts N, Townsend N. A systematic review of associations between non-communicable diseases and socioeconomic status within low- and lower-middle-income countries. J Global Health. 2018;8(2). 10.7189/jogh.08.020409 PMC6076564 30140435

[17] United Nations. World Population Prospects - Population Division. [cited 2022 Nov 23]. https://population.un.org/wpp/

[18] Arista Fernández H, Mundaca Rojas KG, Sosa Flores J, Torres Anaya V. Ley 30021 de Promoción de Alimentación Saludable para niños, niñas y adolescentes. Salud Colectiva. 2018;14:639. 10.18294/sc.2018.1679 30517568

[19] Carrillo-Larco RM, Guzman-Vilca WC, Leon-Velarde F, Bernabe-Ortiz A, Jimenez MM, Penny ME et al. Peru – Progress in health and sciences in 200 years of independence. Lancet Reg Health - Americas. 2022;7(December 2021):100148. 10.1016/j.lana.2021.100148 PMC9904031 36777656

[20] Ministry of Health. Know the advertising warnings (octagons). 2019. [cited 2022 Nov 15]. https://www.gob.pe/1066-ministerio-de-salud-conoce-las-advertencias-publicitarias-octogonos

[21] World Cancer Research Fund International. Policy Actions. https://policydatabase.wcrf.org/level_one?page=nourishing-level-one#step2=2#step3=315

[22] Backholer K, Vandevijvere S, Blake M, Tseng M. Sugar-sweetened beverage taxes in 2018: a year of reflections and consolidation. Public Health Nutr. 2018;21(18):3291–5. 30449291 10.1017/S1368980018003324 PMC10260885

[23] Hernández-Vásquez A, Vargas-Fernández R. Changes in the prevalence of overweight and obesity among Peruvian children under five years before and during the COVID-19 pandemic: findings from a Nationwide Population-based study. Int J Environ Res Public Health. 2022;19(19). 10.3390/ijerph191912390 PMC9565992 36231690

[24] Brero M, Meyer CL, Jackson-Morris A, Spencer G, Ludwig‐Borycz E, Wu D, et al. Investment case for the prevention and reduction of childhood and adolescent overweight and obesity in Mexico. Obes Rev. 2023;24(9):e13595. 37464960 10.1111/obr.13595

[25] Haby MM, Vos T, Carter R, Moodie M, Markwick A, Magnus A, et al. A new approach to assessing the health benefit from obesity interventions in children and adolescents: the assessing cost-effectiveness in obesity project. Int J Obes. 2006;30(10):1463–75. 10.1038/sj.ijo.0803469 17003807

[26] Hayes A, Tan EJ, Lung T, Brown V, Moodie M, Baur L. A new model for evaluation of interventions to prevent obesity in early childhood. Front Endocrinol. 2019;10:132. 10.3389/fendo.2019.00132 PMC6405882 30881347

[27] Kim DD, Silver MC, Kunst N, Cohen JT, Ollendorf DA, Neumann PJ. Perspective and costing in cost-effectiveness analysis, 1974–2018. PharmacoEconomics. 2020;38(10):1135–45. 32696192 10.1007/s40273-020-00942-2 PMC7373843

[28] Sanders GD, Neumann PJ, Basu A, Brock DW, Feeny D, Krahn M, et al. Recommendations for conduct, methodological practices, and reporting of cost-effectiveness analyses: second panel on cost-effectiveness in health and medicine. JAMA. 2016;316(10):1093–103. 27623463 10.1001/jama.2016.12195

[29] Husereau D, Drummond M, Augustovski F, de Bekker-Grob E, Briggs AH, Carswell C, et al. Consolidated Health Economic evaluation reporting standards 2022 (CHEERS 2022) Statement: Updated Reporting Guidance for Health Economic Evaluations. Value Health. 2022;25(1):3–9. 35031096 10.1016/j.jval.2021.11.1351

[30] Wilkinson T, Sculpher MJ, Claxton K, Revill P, Briggs A, Cairns JA, et al. The international decision support Initiative Reference Case for economic evaluation: an aid to Thought. Value Health. 2016;19(8):921–8. 27987641 10.1016/j.jval.2016.04.015

[31] Robinson LA, Hammitt JK, Cecchini M, Chalkidou K, Claxton K, Cropper ML, et al. Reference case guidelines for benefit-cost analysis in Global Health and Development. Harvard University and Bill & Melinda Gates Foundation; 2019.

[32] Prüss-Üstün A, Mathers C, Corvalán C, Woodward A. Assessing the environmental burden of disease at national and local levels: introduction and methods. WHO Environmental Burden of Disease Series, No. 1. World Health Organization. 2003 [cited 2022 Nov 28]. https://www.who.int/publications/i/item/9241546204

[33] Kontis V, Mathers CD, Rehm J, Stevens GA, Shield KD, Bonita R, et al. Contribution of six risk factors to achieving the 25×25 non-communicable disease mortality reduction target: A modelling study. Lancet. 2014;384(9941):427–37. 24797573 10.1016/S0140-6736(14)60616-4

[34] International Agency for Research on Cancer (IARC). Cancer and obesity. [cited 2022 Nov 28]. https://gco.iarc.fr/causes/obesity/home

[35] Foreman KJ, Marquez N, Dolgert A, Fukutaki K, Fullman N, McGaughey M, et al. Forecasting life expectancy, years of life lost, and all-cause and cause-specific mortality for 250 causes of death: reference and alternative scenarios for 2016–40 for 195 countries and territories. Lancet. 2018;392(10159):2052–90. 30340847 10.1016/S0140-6736(18)31694-5 PMC6227505

[36] Menon GR, Singh L, Sharma P, Yadav P, Sharma S, Kalaskar S, et al. National Burden estimates of healthy life lost in India, 2017: an analysis using direct mortality data and indirect disability data. Lancet Global Health. 2019;7(12):e1675–84. 31708148 10.1016/S2214-109X(19)30451-6

[37] Gortmaker SL, Wang YC, Long MW, Giles CM, Ward ZJ, Barrett JL, et al. Three interventions that reduce childhood obesity are projected to save more than they cost to implement. Health Aff. 2015;34(11):1932–9. 10.1377/hlthaff.2015.0631 PMC9627551 26526252

[38] Gortmaker SL, Long MW, Resch SC, Ward ZJ, Cradock AL, Barrett JL, et al. Cost effectiveness of childhood obesity interventions: evidence and methods for CHOICES. Am J Prev Med. 2015;49(1):102–11. 26094231 10.1016/j.amepre.2015.03.032 PMC9508900

[39] World Health Organization. Global Health Expenditure Database. [cited 2022 Nov 23]. https://apps.who.int/nha/database

[40] World Bank. World Development indicators. Published; 2021.

[41] Shekar M, Popkin B, Obesity. Health and Economic consequences of an Impending Global Challenge. Washington: World Bank Group; 2020.

[42] Hagman E, Danielsson P, Brandt L, Svensson V, Ekbom A, Marcus C. Childhood obesity, obesity treatment outcome, and Achieved Education: a prospective cohort study. J Adolesc Health. 2017;61(4):508–13. 28693958 10.1016/j.jadohealth.2017.04.009

[43] OECD. Context and description of the current Peruvian pension system. In: OECD Reviews of Pension Systems: Peru. OECD. 2019 [cited 2023 May 19]. pp. 19–41. (OECD Reviews of Pension Systems). https://www.oecd-ilibrary.org/finance-and-investment/oecd-reviews-of-pension-systems-peru_a62a1bc2-en

[44] Palinkas LA, Horwitz SM, Green CA, Wisdom JP, Duan N, Hoagwood K. Purposeful sampling for Qualitative Data Collection and Analysis in mixed method implementation research. Adm Policy Ment Health. 2015;42(5):533–44. 24193818 10.1007/s10488-013-0528-y PMC4012002

[45] Hsieh HF, Shannon SE. Three approaches to qualitative content analysis. Qual Health Res. 2005;15(9):1277–88. 16204405 10.1177/1049732305276687

[46] WHO. Report of the commission on ending childhood obesity. Geneva; 2016.

[47] Policy Briefs – STOP. [cited 2022 Nov 28]. http://www.stopchildobesity.eu/policy-briefs/

[48] Instituto Nacional de Estadística e Informática. Perú: Encuesta Demográfica y de Salud Familiar-ENDES 2020. Perú; 2021 May [cited 2024 Feb 26] p. 216. https://www.inei.gob.pe/media/MenuRecursivo/publicaciones_digitales/Est/Lib1795/

[49] The Partnership for Healthy Cities. Lima, Peru | Partnership for Healthy Cities COVID-19 Response Center. [cited 2022 Dec 1]. https://cities4health.org/city-stories/lima-peru

[50] The World Bank. World Development Indicators | DataBank. [cited 2022 Nov 23]. https://databank.worldbank.org/source/world-development-indicators

[51] Instituto Nacional de Estadística e Informática. Perú Resultados Definitivos de los Censos Nacionales 2017. Perú; 2018 Oct [cited 2024 Feb 28] p. 32. https://censo2017.inei.gob.pe/resultados-definitivos-de-los-censos-nacionales-2017/

[52] Pradeilles R, Pareja R, Creed-Kanashiro HM, Griffiths PL, Holdsworth M, Verdezoto N et al. Diet and food insecurity among mothers, infants, and young children in Peru before and during COVID-19: a panel survey. Maternal Child Nutr. 2022;18(3). 10.1111/mcn.13343 PMC9115223 35274825

[53] Rollins NC, Bhandari N, Hajeebhoy N, Horton S, Lutter CK, Martines JC, et al. Why invest, and what it will take to improve breastfeeding practices? Lancet. 2016;387(10017):491–504. 26869576 10.1016/S0140-6736(15)01044-2

[54] Torres-Álvarez R, Barrán-Zubaran R, Canto-Osorio F, Sánchez-Romero LM, Camacho-García-Formentí D, Popkin BM et al. Body weight impact of the sugar-sweetened beverages tax in Mexican children: A modeling study. Pediatric Obesity. 2020;15. 10.1111/ijpo.12636 32282131

[55] Lindberg L, Persson M, Danielsson P, Hagman E, Marcus C. Obesity in childhood, socioeconomic status, and completion of 12 or more school years: a prospective cohort study. BMJ Open. 2021;11(3):1–11. 10.1136/bmjopen-2020-040432 PMC7957136 33707266

[56] Jamison DT, Summers LH, Alleyne G, Arrow KJ, Berkley S, Binagwaho A et al. Appendix 3: Global health 2035: a world converging within a generation. Salud publica de Mexico. 2015/11/07 ed. 2015;57(5):444–67. 26545007

[57] Haacker M, Hallett TB, Atun R. On discount rates for economic evaluations in global health. Health Policy Plann. 2019;czz127. 10.1093/heapol/czz127 31625564

[58] Domestic general government health expenditure. (% of current health expenditure). World Bank Open Data; [cited 2023 Dec 30]. https://data.worldbank.org

[59] Hutubessy R, Chisholm D, Edejer TTT, WHO-CHOICE. Generalized cost-effectiveness analysis for national-level priority-setting in the health sector. Cost Eff Resource Allocation. 2003;1(1):8. 10.1186/1478-7547-1-8 PMC320499 14687420

[60] Saavedra-Garcia L, Meza-Hernández M, Yabiku-Soto K, Hernández-Vásquez A, Kesar HV, Mejia-Victorio C, et al. Food and Beverage supply and advertising in schools and their surroundings in metropolitan lima. An exploratory study. Revista Peruana De Med Experimental Y Salud Publica. 2020;37(4):726–32. 10.17843/rpmesp.2020.374.5838 33566915

[61] The World Bank. Improving Maternal and Child Health and Nutrition in Peru. 2017 [cited 2022 Dec 3]. https://www.worldbank.org/en/results/2017/04/07/improving-maternal-child-health-nutrition-peru

[62] Ministerio de Salud. Norma Técnica De Salud Atención integral y diferenciada de la Gestante Adolescente Durante El Embarazo, Parto Y Puerperio. Biblioteca Virtual en Salud-MINSA; 2020.

[63] Wynne SJ, Duarte R, De Wildt G, Meza G, Merriel A. The timing and quality of antenatal care received by women attending a primary care centre in Iquitos, Peru: a facility exit survey. PLoS ONE. 2020;15(3):1–22. 10.1371/journal.pone.0229852 PMC7058332 32134987

[64] Pia Chaparro M, Bernabe-Ortiz A, Harrison GG. Association between food assistance program participation and overweight. Rev Saude Publica. 2014;48(6):889–98. 26039391 10.1590/S0034-8910.2014048005359 PMC4285835

[65] Afshin A, Peñalvo JL, Gobbo L, Del, Silva J, Michaelson M, O’Flaherty M et al. The prospective impact of food pricing on improving dietary consumption: a systematic review and meta-analysis. PLoS ONE. 2017;12(3). 10.1371/journal.pone.0172277 PMC5332034 28249003

[66] Aceves-Martins M, Llauradó E, Tarro L, Moreno-García CF, Escobar TGT, Solà R, et al. Effectiveness of social marketing strategies to reduce youth obesity in European school-based interventions: a systematic review and meta-analysis. Nutr Rev. 2016;74(5):337–51. 27018054 10.1093/nutrit/nuw004 PMC4836715

[67] Cecchini M, Sassi F, Lauer JA, Lee YY, Guajardo-Barron V, Chisholm D. Tackling of unhealthy diets, physical inactivity, and obesity: Health effects and cost-effectiveness. Lancet. 2010;376(9754):1775–84. 21074255 10.1016/S0140-6736(10)61514-0

[68] Holla-Bhar R, Iellamo A, Gupta A, Smith JP, Dadhich JP. Investing in breastfeeding - the world breastfeeding costing initiative. Int Breastfeed J. 2015;10(1):1–12. 25873985 10.1186/s13006-015-0032-y PMC4396713

[69] Bhutta ZA, Das JK, Rizvi A, Gaffey MF, Walker N, Horton S, et al. Evidence-based interventions for improvement of maternal and child nutrition: what can be done and at what cost? Lancet. 2013;382(9890):452–77. 23746776 10.1016/S0140-6736(13)60996-4

[70] Levasseur P. Do junk food bans in school really reduce childhood overweight? Evidence from Brazil. Food Policy. 2021;99(November).

[71] Sassi F. Obesity and the Economics of Prevention: fit not Fat. Paris: OECD Publishing; 2010.

