# Supplementary material for: The case for investment in nutritional interventions to prevent and reduce childhood and adolescent overweight and obesity in Peru: a modelling study
Source: Int J Behav Nutr Phys Act. 2024 Nov 6;21:127. doi: 10.1186/s12966-024-01677-5 (PMC11542222; doi:10.1186/s12966-024-01677-5)
Supplement: Supplementary file 1 — Supplementary Material 1 [file 12966_2024_1677_MOESM1_ESM.docx]

**The case for investment in nutritional interventions to prevent and reduce childhood and adolescent overweight and obesity in Peru: a modelling study - Supplementary material**

**Table S1. Consolidated Health Economic Evaluation Reporting Standards (CHEERS) 2022 Checklist** [1]**.**

| **Section/topic** | **Item** | **Guidance for reporting** | **Reporting in section** |
| --- | --- | --- | --- |
| Title | 1 | Identify the study as an economic evaluation and specify the interventions being compared. | Title |
| **Abstract** | | | |
| Abstract | 2 | Provide a structured summary that highlights context, key methods, results, and alternative analyses. | Abstract |
| **Introduction** | | | |
| Background and Objectives | 3 | Give the context for the study, the study question, and its practical relevance for decision making in policy or practice. | Background |
| **Methods** | | | |
| Health economic analysis | 4 | Indicate whether a health economic analysis plan was developed and where available. | Not reported – Analysis was not a prospective study conducted alongside a clinical trial. |
| Study population | 5 | Describe characteristics of the study population (such as age range, demographics, socioeconomic, or clinical characteristics). | Methods - Baseline scenario |
| Setting and location | 6 | Provide relevant contextual information that may influence findings. | Methods - Baseline scenario and Intervention scenario |
| Comparators | 7 | Describe the interventions or strategies being compared and why chosen. | Methods - Intervention scenario |
| Perspective | 8 | State the perspective(s) adopted by the study and why chosen. | Methods |
| Time horizon | 9 | State the time horizon for the study and why appropriate | Methods |
| Discount Rate | 10 | Report the discount rate(s) and reason chosen. | Methods- Baseline scenario |
| Selection of outcomes | 11 | Describe what outcomes were used as the measure(s) of benefit(s) and harm(s). | Methods-Baseline scenario and non-economic outcomes |
| Measurements of outcomes | 12 | Describe how outcomes used to capture benefit(s) and harm(s) were measured. | Methods-Baseline scenario and non-economic outcomes |
| Valuation of outcomes | 13 | Describe the population and methods used to measure and value outcomes. | Methods-Baseline scenario |
| Measurement and valuation of resources and costs | 14 | Describe how costs were valued. | Methods-Baseline scenario |
| Currency, price date, and conversion | 15 | Report the dates of the estimated resource quantities and unit costs, plus the currency and year of conversion. | Methods-Table 1 |
| Rationale and description of model | 16 | If modeling is used, describe in detail and why used. Report if the model is publicly available and where it can be accessed. | Methods-Baseline scenario |
| Analytics and assumptions | 17 | Describe any methods for analysing or statistically  transforming data, any extrapolation methods, and  approaches for validating any model used. | Assumptions: Methods and Supplementary material  Model Validation: Methods |
| Characterizing heterogeneity | 18 | Describe any methods used for estimating how the results of the study vary for subgroups. | Methods-Baseline scenario |
| Characterizing distributional effects | 19 | Describe how impacts are distributed across different  individuals or adjustments made to reflect priority  populations. | Data needed to disaggregate the impact across priority populations (e.g., sex and socioeconomic status) was unavailable when the analysis was conducted. |
| Characterizing uncertainty | 20 | Describe methods to characterize any sources of uncertainty in the analysis. | Methods and Supplementary material - Sensitivity analysis |
| Approach to engagement with patients and others affected by the study | 21 | Describe any approaches to engage patients or service recipients, the general public, communities, or stakeholders (such as clinicians or payers) in the design of the study. | Methods- Intervention scenario |
| **Results** | | | |
| Study parameters | 22 | Report all analytic inputs (such as values, ranges, references) including uncertainty or distributional assumptions. | Methods – Sensitivity Analysis; Supplementary material |
| Summary of main results | 23 | Report the mean values for the main categories of costs and outcomes of interest and summarize them in the most appropriate overall measure. | Results Tables 1-7 |
| Effect of uncertainty | 24 | Describe how uncertainty about analytic judgments, inputs, or projections affect findings. Report the effect of choice of discount rate and time horizon, if applicable. | Methods – Indicators; Methods – Sensitivity analysis; Results; Supplementary material Tablse S3. |
| Effect of engagement with patients and others affected by the study | 25 | Report on any difference patient/service recipient, general public, community, or stakeholder involvement made to the approach or findings of the study | Methods – Intervention scenario |
| **Discussion** | | | |
| Study findings, limitations, generalizability, and current knowledge | 26 | Report key findings, limitations, ethical or equity  considerations not captured, and how these could affect patients, policy, or practice. | Discussion |
| **Other Relevant Information** | | | |
| Source of funding | 27 | Describe how the study was funded and any role of the funder in the identification, design, conduct, and reporting of the analysis | Title Page - Acknowledgements |
| Conflicts of interest | 28 | Report authors conflicts of interest according to journal or International Committee of Medical Journal Editors requirements. | Title Page - Conflict of Interest Form |

**Table S2: Baseline Level of Interventions in Peru and Effect Size and Cost Data to Reach Target Goals**

| **Intervention** | **Target population** | **Baseline coverage** | **Target coverage** | **Effect size (95% CI)** | **Effect size source** | **Unit cost (cost per child, 2020 USD)** | **Cost components** | **Unit cost source** |
| --- | --- | --- | --- | --- | --- | --- | --- | --- |
| Social marketing in schools | Children and adolescents aged 6-16 years | 0% | 80% of target population | -0.25 (-0.45, -0.04) BMI reduction | Aceves-Martins et al. (2016) [2] | USD 1.04 | Program organization costs, training of teachers and food service staff, extra teaching, and additional curricular activities, such as brochures and books | Cecchini et al. (2010) [3] |
| Breastfeeding promotion at health centers | Mothers of infants aged 6 months or less | 0% | 85% of the target population | Average 5.2% (3.5%, 6.5%) reduction in obesity prevalence at age 5  (With exposure to breastfeeding promotion, the relative risk (RR) of exclusive breastfeeding up to 6 months old was 2.52 (1.39, 4.59). (152% (39%, 359%) increase in exclusive breastfeeding)  Obesity prevalence is reduced by 31% (21%, 39%) among children at 5 years old who were exclusively breastfed from ages 0-6 months.) | Rollins (2016)[4] and Holla-Bhar (2015) [5] | USD 21.57 | Health education to mothers and training health workers and community volunteers | Rollins (2016)[4] and Bhutta et al. (2013) [6] |
| Healthy food school environment | Children and adolescents aged 6-16 | 3.38% | 80% of target population.  As the intervention only reaches those enrolled in primary and secondary schools, the intervention would need to be implemented in 87% of primary and secondary schools across Peru. The intervention is assumed to affect 100% of students enrolled in these schools. According to most recent data available from the World Bank, Peru’s net primary school enrollment rate was 96% in 2018 and net secondary school enrollment rate was 89% in 2018.[7] | Boys - 5.1% (0.9%, 9.3%) reduction in overweight prevalence when junk food is unavailable at school canteens (Significant)  Girls - 1.8% (-2.8%, 6.4%) reduction in overweight prevalence when junk food is unavailable at school canteens (Not significant) | Levasseur (2021) [8] | USD 0.19 | Basic administration, planning, enforcement, preparation and distribution of posters, and monitoring | Sassi (2010) [9] |
| 20% food subsidy | Children and adolescents aged 0-19 years | 0% | 20.2% of target population (Figure based on proportion of children and adolescents living below the poverty line). | -0.08 (-0.16, 0.00) BMI reduction | Afshin et al. (2017) [10] | USD 0.02 | Planning and development, operations, administration, and monitoring | Sassi (2010) [9] |

**Future BMI projections**

For the model cohort, we projected future BMI for every year from ages 5 to 19 and then in 5-year increments after 20 years old [11,12]. We used single-year age groups for children and adolescents because the relationship between BMI and overweight and obesity is more variable during this period and becomes more stable in adulthood, where we used 5-year age groups. For this analysis, adult overweight is considered a body mass index (BMI, calculated as weight in kilograms divided by the square of height in meters) of 25 to <30 and obesity is a BMI of 30 and above, while for children, overweight is a BMI-for-age above one standard deviation of the World Health Organization (WHO) Growth Reference median for children of the same age and sex, and obesity is a BMI-for-age above two standard deviations of the median [13]. To do this, we used cohort and age effects on BMI using data for Peru from the NCD-Risk Factor Collaboration, estimated using multiple linear regression [11,12]. Mean BMI was then converted into the prevalence of overweight and obesity. The relationship was modeled separately by sex and single-year age group for children and adolescents 5-19 years old, and by sex and five-year age group for adults 20 and above, to account for the changing relationship between BMI and overweight and obesity through the life course. The relationship between BMI and overweight and obesity prevalence is non-linear for ages between 5 to 19; to account for this, we modeled the relationship between BMI and overweight prevalence and obesity prevalence using a cubic spline of mean BMI, with knots at every 5 years [14]. For adults 20 years and over, we used a linear regression to project future overweight and obesity prevalence of the model cohort [15,16].

**Obesity-attributable healthcare costs**

Data is only available on the additional healthcare cost for individuals affected by obesity; therefore, the analysis includes only the additional healthcare costs of obesity (excluding overweight). To obtain the additional healthcare costs attributable to childhood and adolescent obesity, we use the incremental annual healthcare expenditure for a person affected by obesity. This was calculated by multiplying the average annual healthcare expenditure per person by the incremental percentage higher healthcare expenditure paid by individuals affected by obesity (as compared to that paid by those of healthy weight), disaggregated by age [17], since healthcare costs attributable to obesity change during the life cycle [18]. We used the average annual healthcare expenditure in Peru for 2019 from the WHO Global Health Expenditure Database. Data on age-specific healthcare expenditure are not available for Peru, so we assume the same annual expenditure for all ages. For the incremental percentage higher healthcare expenditure paid by those affected by obesity, we relied on a study in the United States, which found that compared to individuals with healthy weight, there was no significant difference in healthcare expenditure incurred by individuals who are affected by obesity under six years; however, at age 6-19, individuals affected by obesity incur 35.9% higher healthcare expenditure, and this increases to 21.2% at age 20 and further to 33.3% at age 73 years and older [17]. We did not estimate the additional healthcare cost of overweight only, as no data is available on the additional healthcare cost of overweight only. We acknowledge this will underestimate the total economic costs of childhood and adolescent obesity and overweight. Thus, the obesity-attributable healthcare cost for age-group *a* in year *y* was calculated using the formula: 
 *Obesity attributable healthcare cost_a,y_*=$\frac{N*C}{{(1+r)}^{(y-2025)}}$
Where *N* is the number of individuals affected by obesity, *C* is the additional annual obesity-attributable healthcare cost per person.

**Impact on labor productivity**

The lifetime loss in wages was modeled for lower education attainment and productivity loss due to absenteeism and presenteeism attributable to childhood overweight and obesity. To estimate the impact on educational attainment, the number of individuals affected by obesity at age 17 (the age when individuals in Peru complete secondary education to begin tertiary education) and the proportion of the Peruvian population aged 25-34 with tertiary education, were used to estimate the number of individuals who would not attain tertiary education due to overweight and obesity [19]. The lifetime loss in wages was then calculated as:

$${Lifetime loss in wages}_{a}={[N}_{a}w(u_{tert}-u_{sec})(65-22)]-[4N_{a}wu_{sec}]$$

Where *N_a_* is the number of individuals in sub-cohort *a* who did not attain tertiary education due to obesity, calculated using prevalence of obesity at age 17, percentage of 25-34-year-olds with tertiary education in Peru obtained from INEI [20], and the OR of completing ≥12 years of education; *w* is the labor force participation rate; *u_tert_* is the expected annual wages with tertiary education; and *u_sec_* is the expected annual wages with secondary education. Labor force participation rate, disaggregated by sex, are obtained from the World Development Indicators [7], and annual wages, by education attainment and sex, are obtained from Instituto Nacional de Estadística e Informática [20]. We accounted for the five additional years during which individuals who did not attend tertiary school may have worked (since tertiary education lasts for five years in Peru) and assumed that every individual who attained tertiary education would enter the workforce at age 22 and exit at age 65. The lifetime wages lost by each sub-cohort *a* was discounted at 3% annually. Obesity is also associated with lower work productivity in terms of absenteeism and presenteeism [21]. As data from Peru is not currently available, a systematic review was used to estimate the productivity loss due to obesity-related absenteeism and presenteeism.

**Potential impact fraction (PIF)**

PIF is the proportional change in mortality and morbidity attributable to a change in exposure to a risk factor due to implementation of the interventions in a population. For overweight and obesity, PIF for each overweight and obesity-attributable condition is calculated as:

  $PIF=\frac{{\sum_{j}P_{j}{RR}_{j}-\sum_{j}\hat{P}_{j}{RR}_{j}}}{\sum_{j}P_{j}{RR}_{j}}$

Where *P_j_* is population distribution of each BMI category *j* (healthy, overweight, and obese) in the baseline scenario, is the population distribution of BMI category *j* in the intervention scenario, and *RR_j_* are the relative risks of mortality and/or morbidity due to each overweight and obesity-attributable condition for each BMI category *j* obtained from GBD 2019 and from Rajan and Menon (2017) for depression [9]. We calculated the PIFs for each obesity-attributable disease/condition at every age of the cohort from 2026 (the year when the first young people in the cohort would turn 20) to 2092. The PIFs, by cause, age, and sex, were multiplied by the baseline YLLs and YLDs to obtain YLLs and YLDs saved due to the intervention.

**Sensitivity Analyses**

To test the sensitivity of the results to changes in our assumptions, we conducted additional analyses. First, we doubled the time for the full effects of the interventions to be realized from one to two years following implementation. A second sensitivity analysis varied the impact of childhood and adolescent obesity on educational attainment. Based on the same cohort used in the study of Hagman et al. [19], after controlling for migration background, attention deficit disorder, anxiety, depression, and parental socioeconomic status, Lindberg et al. found that obesity in childhood is associated with a 43% lower likelihood of not completing 12 or more years of education [22]. We also conducted sensitivity analyses to consider how the changes in national income (GDP per capita) impacted the value of these changes in mortality or life expectancy. Using the Lancet Commission on Investing in Health (CIH)’s GDP multipliers developed based on life year valuation estimates from life expectancy changes between 2000 and 2011, we estimated an upper bound to the economic value of premature mortality [23,24]. Finally, as there is a growing discussion around using a higher discount rate in low and middle-income countries to illustrate a preference for receiving benefits earlier, we have also conducted an additional sensitivity analysis that applies a higher discount rate of 5% [25].

**Table S3. Sensitivity analyses for the return on investment (ROI) of selected childhood and adolescent obesity interventions**

| Sensitivity Analysis Scenarios | ROI (95% CI) | | |
| --- | --- | --- | --- |
|  | Over 30 years | Over 50 years | Over lifetime |
| Scenario 1: effects realized two years after implementation | | | |
| Social marketing in schools | 2.6 (-0.4, 5.5) | 5.7 (0.1, 11.0) | 16.9 (1.9, 31.2) |
| Breastfeeding promotion at health centers | 45.2 (28.4, 58.8) | 77.2 (48.9, 99.9) | 282.8 (179.5, 365.4) |
| Healthy food school environment | 67.7 (9.8, 125.6) | 110.6 (16.7, 204.5) | 304.3 (46.8, 561.8) |
| 20% food subsidy | 112.0 (-1.0, 225.0) | 209.4 (-1.0, 419.7) | 543.6 (-1.0, 1,088.2) |
| All four combined | 10.6 (1.1, 19.9) | 18.7 (2.5, 34.5) | 53.3 (9.1, 96.6) |
| Scenario 2: varying the impact of childhood obesity on education attainment | | | |
| Social marketing in schools | 3.4 (-0.3, 6.8) | 7.2 (0.3, 13.8) | 19.2 (2.2, 35.4) |
| Breastfeeding promotion at health centers | 58.2 (39.1, 73.4) | 152.6 (103.0, 192.2) | 428.1 (289.7, 538.8) |
| Healthy food school environment | 355.5 (301.8, 409.2) | 573.6 (483.6, 663.6) | 1,346.7 (1,111.4, 1,582.0) |
| 20% food subsidy | 81.5 (-12.1, 176.6) | 154.2 (-21.9, 333.3) | 380.6 (-52.4, 820.9) |
| All four combined | 42.9 (42.7, 43.2) | 71.7 (71.2, 72.1) | 171.3 (170.2, 172.4) |
| Scenario 3: valuing one life-year as GDP per capita times global GDP multiplier (1.6) | | | |
| Social marketing in schools | 3.6 (-0.3, 7.1) | 9.4 (0.6, 17.3) | 26.4 (3.3, 47.6) |
| Breastfeeding promotion at health centers | 61.3 (41.2, 77.4) | 174.3 (117.8, 219.6) | 547.1 (370.3, 688.6) |
| Healthy food school environment | 366.9 (311.0, 422.8) | 733.2 (620.4, 846.0) | 1,797.9 (1,487.0, 2,108.7) |
| 20% food subsidy | 85.3 (-12.6, 184.9) | 193.9 (-27.2, 418.8) | 510.1 (-69.8, 1,099.9) |
| All four combined | 44.4 (44.1, 44.6) | 91.3 (90.7, 91.9) | 228.6 (227.1, 230.2) |
| Scenario 4: valuing one life-year as GDP per capita times regional GDP multiplier for Latin America (1.4) | | | |
| Social marketing in schools | 3.4 (-0.3, 6.8) | 8.4 (0.5, 15.6) | 23.7 (2.9, 42.9) |
| Breastfeeding promotion at health centers | 58.8 (39.5, 74.2) | 159.6 (107.8, 201.0) | 497.4 (336.6, 626.0) |
| Healthy food school environment | 353.0 (299.1, 406.8) | 661.5 (559.3, 763.7) | 1,628.3 (1,346.0, 1,910.5) |
| 20% food subsidy | 81.6 (-12.1, 176.9) | 175.1 (-24.7, 378.3) | 460.7 (-63.2, 993.5) |
| All four combined | 42.6 (42.4, 42.9) | 82.4 (81.8, 82.9) | 207.0 (205.6, 208.3) |
| Scenario 5: using an annual discounting rate of 5% | | | |
| Social marketing in schools | 1.7 (-0.6, 3.8) | 4.0 (-0.2, 8.0) | 12.9 (1.2, 24.1) |
| Breastfeeding promotion at health centers | 33.6 (22.4, 42.5) | 81.3 (54.8, 102.6) | 261.3 (176.7, 329.0) |
| Healthy food school environment | 223.4 (190.0, 256.7) | 361.4 (306.6, 416.3) | 944.8 (785.9, 1,103.7) |
| 20% food subsidy | 55.3 (-8.6, 120.2) | 104.3 (-15.2, 225.9) | 289.4 (-40.1, 624.4) |
| All four combined | 29.5 (29.3, 29.6) | 49.3 (49.0, 49.6) | 131.8 (130.9, 132.6) |
| Scenario 6: reducing impact of each intervention by 50% | | | |
| All four combined | 33.7 (29.2, 38.0) | 54.8 (47.2, 62.2) | 137.1 (116.3, 157.4) |
| Scenario 7: increasing the impact of each intervention by 10% | | | |
| All four combined | 40.3 (30.6, 49.7) | 66.4 (49.6, 82.7) | 168.9 (123.2, 213.6) |

**Exhibit S1.Peru Childhood and Adolescent Overweight and Obesity Investment Case Key Informant Interview Guide**

*Thank interviewee for their time and participation.*

*Describe project, anticipated outputs, and how interviewee’s participation will contribute to the project.*

The purpose of this project is to develop an investment case on preventing and reducing overweight and obesity among children and adolescents in Peru. This includes supporting UNICEF in identifying available evidence on interventions and selecting the most appropriate interventions for inclusion in the investment case. It also includes research on the institutional context within the country to understand the diverse range of stakeholders relevant to the prevention and control of overweight and obesity among children and adolescents. Your input will be instrumental in helping us understand the context of childhood and adolescent overweight obesity policy in Peru and in identifying the most appropriate interventions for Peru. These interventions may be new to Peru or may be already existing policies and programs that could be expanded or strengthened.

*Ask for permission to record interview.*

We would like to record this interview to supplement our note taking.

We will not quote you or attribute to you anything you say during the interview without first seeking your permission.

*Intervention Selection Questions*

1. Please briefly describe your experience working with childhood and adolescent overweight and obesity and the specific interventions or policies that you have been a part of?
2. Thinking about the childhood and adolescent overweight and obesity interventions or policies that you have been involved with, or are aware of, can you describe some of the key successes? How were these evaluated? What factors do you think were most important in achieving those successes?
3. Can you describe some of the key barriers that were encountered?
4. What strategies were employed to overcome these barriers?
5. Have any interventions or policies related to childhood and adolescent overweight and obesity not been successful, and if so, can you please explain why?
6. If Peru has implemented any policies designed to reduce overweight and obesity prevalence, are there any changes to existing national policies that you would recommend to increase their effectiveness?
7. Which policies or interventions do you believe could be the most cost-effective and feasible given the political and economic context in Peru?
8. Generally, what capacity gaps or areas for strengthening should be considered when selecting policies that address Peru’s context? (For example, if a country were considering implementing a tax on sugar sweetened beverages, the capacity to collect tax revenue, monitoring and enforcement structures and capacity, and ability to counter private sector opposition should all be considered.)
9. Are there any municipal or state level policies that you believe should be implemented at national level?
10. What communication or dissemination strategies and messages are most likely to be successful for different audiences for the policies/interventions you identified above? For example, how might different strategies or policies be employed for:

- Policymakers
- Politicians
- The public
- Private industry?

1. Who do you see as the most important private sector actors and how do you think they are likely to respond to policy reforms aimed at reducing childhood and adolescent overweight and obesity?

Is there anything else you would like to share regarding childhood and adolescent overweight and obesity in Peru and the selection of interventions that should be included in a childhood and adolescent overweight and obesity investment case model that has not been covered in these questions?

*Situational Analysis Questions*

1. Peru’s overweight and obesity prevalence rates have grown rapidly over the past few years, with especially fast growth seen among children. What macroeconomic and social trends in Peru have led to the current level of overweight and obesity prevalence?
2. In Peru, are there agencies and organizations that implement or influence childhood and adolescent overweight and obesity interventions and policies?
   - How would you describe the quality and degree of coordination between the different agencies and organizations?
   - Are there any specific areas of coordination that are particularly difficult, or requiring improvement?
   - What do you see as the main barriers to better coordination and communication and how might these be addressed?
3. What do you think will be the public response, or different responses from different segments of the public to policy reforms aimed at reducing childhood and adolescent overweight and obesity?
4. What mechanisms are currently in place to finance overweight and obesity prevention and what additional opportunities exist? For example, earmarking revenue from sugar sweetened beverage taxes for overweight and obesity prevention.
5. What wider implementation capacities are available that would facilitate the implementation of overweight and obesity prevention policies? For example, the ability to coordinate across sectors or a well-trained, affordable, and accessible primary healthcare system.
6. In relation to policies or interventions that prevent child and adolescent overweight and obesity, which do you think are most likely to be cost-effective and feasible in Peru – economically and politically ?:
7. How might Peru’s previous experiences with major policy reforms inform how these interventions or policies would be received?
8. What would you anticipate as the main obstacles?
9. What communication or dissemination strategies and messages are most likely to be successful?
10. Is there anything else you would like to share regarding the political and policy landscape and the opportunities and challenges that has not been covered in these questions?

**References**

1. Husereau D, Drummond M, Augustovski F, de Bekker-Grob E, Briggs AH, Carswell C, et al. Consolidated Health Economic Evaluation Reporting Standards 2022 (CHEERS 2022) Statement: Updated Reporting Guidance for Health Economic Evaluations. Value Health. 2022 Jan;25(1):3–9.

2. Aceves-Martins M, Llauradó E, Tarro L, Moreno-García CF, Escobar TGT, Solà R, et al. Effectiveness of social marketing strategies to reduce youth obesity in European school-based interventions: A systematic review and meta-analysis. Nutrition Reviews. 2016;74(5):337–51.

3. Cecchini M, Sassi F, Lauer JA, Lee YY, Guajardo-Barron V, Chisholm D. Tackling of unhealthy diets, physical inactivity, and obesity: Health effects and cost-effectiveness. The Lancet. 2010;376(9754):1775–84.

4. Rollins NC, Bhandari N, Hajeebhoy N, Horton S, Lutter CK, Martines JC, et al. Why invest, and what it will take to improve breastfeeding practices? The Lancet. 2016;387(10017):491–504.

5. Holla-Bhar R, Iellamo A, Gupta A, Smith JP, Dadhich JP. Investing in breastfeeding - the world breastfeeding costing initiative. International Breastfeeding Journal. 2015;10(1):1–12.

6. Bhutta ZA, Das JK, Rizvi A, Gaffey MF, Walker N, Horton S, et al. Evidence-based interventions for improvement of maternal and child nutrition: What can be done and at what cost? The Lancet. 2013;382(9890):452–77.

7. The World Bank. World Development Indicators | DataBank [Internet]. [cited 2022 Nov 23]. Available from: https://databank.worldbank.org/source/world-development-indicators

8. Levasseur P. Do junk food bans in school really reduce childhood overweight? Evidence from Brazil. Food Policy. 2021;99(November).

9. Sassi F. Obesity and the Economics of Prevention: Fit not Fat. Paris: OECD Publishing; 2010.

10. Afshin A, Peñalvo JL, Gobbo L Del, Silva J, Michaelson M, O’Flaherty M, et al. The prospective impact of food pricing on improving dietary consumption: A systematic review and meta-analysis. PLoS ONE. 2017;12(3).

11. Bentham J, Di Cesare M, Bilano V, Bixby H, Zhou B, Stevens GA, et al. Worldwide trends in body-mass index, underweight, overweight, and obesity from 1975 to 2016: a pooled analysis of 2416 population-based measurement studies in 128·9 million children, adolescents, and adults. The Lancet. 2017;390(10113):2627–42.

12. Haby MM, Vos T, Carter R, Moodie M, Markwick A, Magnus A, et al. A new approach to assessing the health benefit from obesity interventions in children and adolescents: The assessing cost-effectiveness in obesity project. International Journal of Obesity. 2006;30(10):1463–75.

13. Arts M, Bégin F, Aguayo V. Prevention of overweight and obesity in children and adolescents: UNICEF programming guidance. Published online August 2019;

14. Stevens GA, Singh GM, Lu Y, Danaei G, Lin JK, Finucane MM, et al. National, regional, and global trends in adult overweight and obesity prevalences. Population Health Metrics. 2012;10:1–16.

15. Lobato JCP, Kale PL, Velarde LGC, Szklo M, Costa AJL. Correlation between mean body mass index in the population and prevalence of obesity in Brazilian capitals: Empirical evidence for a population-based approach of obesity Disease epidemiology - Chronic. BMC Public Health. 2015;15(1):1–6.

16. Armitage P, Berry G. Statistical Methods in Medical Research. 3rd ed. Blackwell; 1994.

17. Gortmaker SL, Wang YC, Long MW, Giles CM, Ward ZJ, Barrett JL, et al. Three interventions that reduce childhood obesity are projected to save more than they cost to implement. Health Affairs. 2015;34(11):1932–9.

18. Gortmaker SL, Long MW, Resch SC, Ward ZJ, Cradock AL, Barrett JL, et al. Cost Effectiveness of Childhood Obesity Interventions: Evidence and Methods for CHOICES. American Journal of Preventive Medicine. 2015;49(1):102–11.

19. Hagman E, Danielsson P, Brandt L, Svensson V, Ekbom A, Marcus C. Childhood Obesity, Obesity Treatment Outcome, and Achieved Education: A Prospective Cohort Study. Journal of Adolescent Health. 2017;61(4):508–13.

20. Instituto Nacional de Estadística e Informática. PERÚ Instituto Nacional de Estadística e Informática [Internet]. [cited 2022 Nov 23]. Available from: https://www.inei.gob.pe/

21. Shekar M, Popkin B. Obesity: Health and Economic Consequences of an Impending Global Challenge. Washington: World Bank Group; 2020.

22. Lindberg L, Persson M, Danielsson P, Hagman E, Marcus C. Obesity in childhood, socioeconomic status, and completion of 12 or more school years: A prospective cohort study. BMJ Open. 2021;11(3):1–11.

23. Okunogbe A, Nugent R, Spencer G, Ralston J, Wilding J. Economic impacts of overweight and obesity: Current and future estimates for eight countries. BMJ Global Health. 2021;6(10).

24. Jamison DT, Summers LH, Alleyne G, Arrow KJ, Berkley S, Binagwaho A, et al. Global health 2035: A world converging within a generation. The Lancet. 2013;382(9908):1898–955.

25. Haacker M, Hallett TB, Atun R. On discount rates for economic evaluations in global health. Health Policy and Planning. 2020;35(1):107–14.
